# An Unusual Case

**Published:** 1885-07

**Authors:** 


### An Unusual Case.

A correspondent in a recent letter says: "I have a peculiar case, upon which I wish some advice. The patient, is Mr. B., age twenty-five years, lymphatic temperament; he was salivated four years ago, the gums on one side, both above and below have been swollen ever since, to such an extent that the crowns of the teeth are well nigh covered. The gums are not painful to the touch, are very hard, and the swelling extends from the median line, to the third molars. I have given local treatment, principally iodine in some form, have also administered iodide of potassium, twelve to fifteen grains a day. At first, this treatment seemed to make some improvement, but very little change has since been made. What further can be done?"

From the above description, it is not an easy matter to make a diagnosis; the description given would seem to indicate that there was enlargement of the alveolus; this might be a hypertrophid condition, from which no active disease would arise: in that case, unless there was a persistent increase of volume, it would perhaps be best to give no treatment, but if the case consists of a thickened indurated condition of the gum, and there is no soreness or pain, and no diseased appearance of the mucous membrane, the case would still be a perplexing one, and perhaps the treatment given is as likely to be efficient as anything else. For hypertrophid gums, excision is frequently resorted to, but from the brief description of this case, and without examination, or at least more thorough knowledge of the case, I would not advise excision. The case is certainly a very interesting one, and we shall hope to hear more about it.

